# Effect of poultry litter amended with biochar or zeolite on nutrient availability, fruit quality, and yield of acid lime in calcareous sandy soil

**DOI:** 10.1038/s41598-026-48057-6

**Published:** 2026-04-20

**Authors:** Shimaa Hosny Gaber, Abu El-Eyuoon Abu Zied Amin, Mohamed F. A. Farghly, Abdallah M. Barakat, Khaled Ahmed Farghly

**Affiliations:** 1 Central Laboratory of Organic Agriculture, Agriculture Research Center, Giza, 12619 Egypt; 2 Soils and Water Department, Faculty of Agriculture, Assiut University, P.O. Box: 71526, Assiut, Egypt; 3 Poultry Production Department, Faculty of Agriculture, Assiut University, P.O. Box: 71526, Assiut, Egypt

**Keywords:** Acid lime, Available nutrients, Biochar, Drip irrigation, Juice weight, Vitamin C, Zeolite, Ecology, Ecology, Environmental sciences, Plant sciences

## Abstract

In the current study, a field experiment of two years (2023 and 2024) was conducted on acid lime (*Citrus aurantifolia* Swingle) to evaluate the effect of poultry litter (PL) amended with biochar (B) or zeolite (Z) in combination with mineral nitrogen fertilizer (MNF) on nutrient availability, growth, yield, and fruit quality of acid lime. This experiment was divided into five treatments, which included: (1) 50% of the recommended nitrogen (N) rate through MNF (50%MNF), (2) 100% of the recommended N rate through MNF (100%MNF), (3) 50% of the recommended N rate through MNF + poultry litter (PL + 50%MNF), (4) 50% of the recommended N rate through MNF+ biochar-amended poultry litter (BPL + 50%MNF), and (5) 50% of the recommended N rate through MNF + zeolite-amended poultry litter (ZPL + 50%MNF). In the first season, applying ZPL + 50%MNF significantly increased soil available nitrogen compared to the 100%MNF treatment. Soil available potassium significantly increased in the first season when BPL + 50%MNF and ZPL + 50%MNF were applied, compared with the rest of the treatments. Significant increase in soil available phosphorus in the first season with applying BPL + 50%MNF compared to the 50%MNF, 100%MNF, and PL treatments. The highest acid lime yields were obtained from the BPL + 50% MNF and ZPL + 50% MNF treatments, recording 15.41 and 15.84 ton/ha, and 16.10 and 16.26 ton/ha in the first and second seasons, respectively. The highest values of total soluble solids, total acidity, and vitamin C content were observed when adding the BPL + 50%MNF and ZPL + 50%MNF treatments during two seasons. Based on these findings, the study suggests that the application of 10 kg of poultry litter amended with biochar or zeolite and 0.5 kg N as ammonium nitrate per acid lime tree enhances nutrient availability and fruit yield of acid lime while reducing reliance on nitrogen chemical fertilizers. It recommends considering poultry litter amended with biochar or zeolite for soil reclamation and as an alternative to traditional fertilizers.

## Introduction

Citrus fruits, which are extensively grown in tropical and subtropical regions, are very important among tree fruits. As perennial crops, they need sufficient nourishment to grow to their full potential^1^. Members of the Rutaceae family, such as the acid lime (*Citrus aurantifolia* Swingle), have gained international recognition for their nutritional advantages, particularly their high vitamin C content. In addition to being consumed, lime fruits are used as raw materials in a variety of businesses and pharmaceutical products^2^. The fruit’s interior quality is reflected in its chemical composition; lime plants require a lot of nutrients because they grow quickly. Supplement fertilizers have a direct impact on citrus production and growth, meeting both internal and external factors, and meeting the world’s fruit demands by promoting tree development^3^.

Egypt faces a land resource issue and a growing need to expand agricultural areas to support its population. Most potential expansion areas are sandy soils with poor properties^4^. Rapid soil organic carbon loss and low nitrogen use efficiency limit crop production in new sandy soils. High nitrogen fertilizers use boosts productivity but risks loss through leaching or volatilization. Organic amendments are essential to improve soil properties, enhance nitrogen use efficiency, and reduce organic carbon decomposition^5,6^. Optimizing nitrogen (N) fertilization is essential for sustaining crop productivity while minimizing environmental risks such as nitrous oxide (N₂O) emissions and nutrient leaching, both of which are influenced by N management strategies in agricultural soils^7^. Specific N management practices can modulate N₂O emissions and nutrient dynamics, highlighting the need for more efficient fertilization approaches^8^.

Litter plays a crucial role in poultry performance and health, and can have an environmental impact after use in the poultry facility, such as serving as a soil amendment^9^. Biochar and zeolite were applied as additives to poultry litter from wheat straw in one study to evaluate their effects on growth performance and litter quality^10^. Zeolites, which are hydrated aluminosilicates of alkaline and alkaline earth cations^11^. These minerals possess several remarkable properties that make them highly valuable for agricultural applications, including a high cation exchange capacity, surface area, and water-holding capacity^12^. Applying zeolite to poultry litter can enhance the chemical, microbiological, and physical integrity of the litter^13^. The addition of zeolite has improved the physical properties of sandy soils by increasing total porosity and water-holding capacity, as well as decreasing bulk density^14,15^. Also, zeolite application to sandy loam soil increased cation exchange capacity^16^. Biochar is a low-cost, carbon-rich material that has emerged as an alternative for many agricultural applications, including use as a poultry litter amendment^17^. The surface area of biochar is several thousand times greater than that of its original, unpyrolyzed feedstock^18^. Biochar represents a promising and innovative approach in poultry farming. Incorporating it into poultry litter composting helps reduce ammonia emissions to the environment and minimizes ammonia toxicity to microorganisms^19^. The addition of these amendments to poultry litter aimed to improve nutrient retention and reduce ammonia volatilization during the rearing period. In this context, incorporating organic amendments such as biochar and zeolite into fertilization programs offers a promising strategy to enhance N retention, reduce N losses, and improve nutrient use efficiency. Biochar has been shown to retain phosphorus through adsorption mechanisms, and its nutrient retention capacity can be further enhanced through surface modifications^20^. Therefore, integrating biochar or zeolite-amended organic amendments with reduced rates of mineral N fertilizer may simultaneously improve soil nutrient availability, crop yield, and environmental sustainability. Research on the use of poultry litter amended with biochar or zeolite in combination with mineral nitrogen fertilizer on crop production, particularly in fruit trees, remains very limited. In particular, studies evaluating its effects on acid lime yield and soil fertility in calcareous sandy soil under a drip irrigation system are extremely scarce and nearly absent in the literature, representing the first field evaluation under calcareous sandy soil conditions. Therefore, we wanted to evaluate the performance of applying poultry litter, biochar-amended poultry litter, and zeolite-amended poultry litter on nutrient soil availability, as well as growth, yield, and fruit quality of acid lime (*Citrus Aurantifolia* Swingle) trees compared to mineral fertilization treatment under calcareous sandy soil. In this study, we hypothesize that adding biochar or zeolite to poultry litter will improve litter quality and poultry rearing conditions, as well as to produce nutrient-rich litter suitable for agricultural use as a soil amendment and that the application of poultry litter amended with biochar or zeolite would enhance nutrient availability in calcareous sandy soil, leading to improved yield and fruit quality of acid lime under a drip irrigation system.

## Materials and methods

### Preparation of poultry litter amended with biochar or zeolite

The poultry litter was prepared by incorporating biochar or zeolite into the bedding material before the rearing period. The letter consisted of dry wheat straw (thickness, 6–8 cm), which was thoroughly mixed with biochar or zeolite at a 10% (w/w) level based on the dry weight of the bedding. The prepared mixture was then evenly spread on the poultry house floor at a thickness of approximately 6–8 cm. Both types of poultry litter were prepared as described by Farghly et al.^8^. After the rearing cycle, the used litter was collected, referred to as biochar-amended poultry litter and zeolite-amended poultry litter, and stored for subsequent use in soil application. The important properties of poultry litter, biochar-amended poultry litter, and zeolite-amended poultry litter are presented in Table 1.

**Table 1:** Some important properties of poultry litter, biochar-amended poultry litter, and zeolite-amended poultry litter (Data were mean ± standard deviation).

Property	Unit	Poultry litter	Biochar-amended poultry litter	Zeolite-amended poultry litter
pH (1: 10 suspension)	–	6.52 ± 0.02	7.32 ± 0.01	7.40 ± 0.01
EC (1: 10 extract)	dS/m	5.15 ± 0.07	4.25 ± 0.07	4.70 ± 0.00
OC	g/kg	502.91 ± 0.00	510.17 ± 2.06	450.58 ± 4.11
Total N	g/kg	25.32 ± 0.66	24.23 ± 1.77	23.29 ± 1.55
Total P	g/kg	5.62 ± 0.27	7.71 ± 2.87	6.40 ± 1.76
Total K	g/kg	14.90 ± 0.31	17.79 ± 1.58	18.38 ± 1.12

*EC* electrical conductivity, *OC* organic carbon, *N* nitrogen, *P* phosphorus, *K* potassium.

### Experimental design

This experiment was carried out at the experimental Farm of the Arab El-Awammer Research Station, Agric. Res. Center (ARC), Assiut Governorate, Egypt, which is located between latitudes 27°, 11′ N, longitudes 31°, 06′ E, and 71 m above sea level, the study was carried out on acid lime (*Citrus Aurantifolia* Swingle) trees during the 2023 and 2024 seasons. The soil under study is characterized as calcareous sandy. For this experiment, fifteen trees that were planted 4 × 4 m apart, were of similar stature and vigor, and showed no indication of nutrient deficit, were divided into five treatments, each with three replicates. Each replication was represented by just one tree. Plants were irrigated using a drip irrigation method. Each tree row had a single drip line, and each tree had two emitters (16 L h^− 1^). The weather (temperature, humidity, etc.) and the trees’ stage of growth affect how much water is needed overall. Poultry litter and biochar- and zeolite-amended poultry litter were added once (10 kg) per tree per year at the end of December. These amendments were added in a trench around the tree, 100 cm away from the tree, in both seasons. Nitrogen was applied at two levels corresponding to 50% and 100% of the recommended rate, using ammonium nitrate (33.5% N) as the nitrogen source. The full rate (100%) was equivalent to 1 kg N per tree, while the reduced rate (50%) corresponded to 0.5 kg N per tree. The nitrogen fertilizer was applied in three equal doses at three stages: at the start of growth, after full bloom, and one month later. All trees received yearly about 450 g K_2_O per tree as potassium sulfate (48–52% K_2_O), divided into two equal doses: late March and mid-August, as well as 150 g P_2_O_5_ per tree as calcium superphosphate (15.5% P_2_O_5_) in one dose at the beginning of the growing season. Chemical fertilizers used in this study were added in a trench around the tree, 100 cm away from the tree, in both seasons. The trees were subjected to the same irrigation and pest control practices that normally occur in an orchard. The experimental trees were divided into five groups, which included: (1) fertilization at 50% of the recommended N rate through mineral N (50%MNF), (2) fertilization at 100% of the recommended N rate through mineral N (100%MNF), (3) fertilization at 50% of the recommended N rate through mineral N + poultry litter (PL + 50%MNF), (4) Fertilization at 50% of the recommended N rate through inorganic N + biochar-amended poultry litter (BPL + 50%MNF), and (5) Fertilization at 50% of the recommended N rate through mineral N + zeolite-amended poultry litter (ZPL + 50%MNF).

### Soil chemical analysis

Soil samples were collected after the harvest of the acid lime fruits in both seasons from a depth of 0–30 cm. The samples were air-dried, crushed, passed through a 2 mm mesh sieve, and stored for chemical analysis. Available nitrogen in soil samples was extracted by 1 M KCl^21^. The available nitrogen in soil extracts was determined by the Kjeldahl method^22^. Available phosphorus (Olsen-P) in the soil samples was extracted by 0.5 M NaHCO_3_ at pH 8.5^23^. Phosphorus in the extracts was measured by colorimetric analysis using the chlorostannous phosphomolybdic acid method^24^. Available potassium in soil samples was extracted with 1 M ammonium acetate, pH 7, and then measured by a flame photometer^25^.

### Vegetative growth

For each replication, four spring growth branches were randomly selected from each tree. The North [N], East [E], South [S], and West [W] branches were chosen, and they were tagged to measure parameters of growth, such as length of branches (cm) and the number of leaves per branch. From each of the four branches, twenty mature mid-branch leaves were sampled (5 leaves each), measured for length and width, and the leaf length/width ratio was computed. The following formula was used to estimate leaf area (cm^2^): Leaf area = 2/3 x length x breadth, as stated by Chou^26^. The amount of chlorophyll in leaves was measured by A chlorophyll meter (SPAD–502Plus, Japan).

### Flowering and fruit set

Two branches from each tree were tagged in two distinct directions (northeast and southwest). For every season, the number of blooms on each branch was noted. Numbers and percentages of setting fruits were counted and computed during both experimental seasons.

The fruit set % equation looked like this:

$${\text{Fruit set percentage}}={\text{(Number of fruit set/Total number of flowers)}}\times 100.$$

### Yield parameters

The total amount of fruit produced by each tree was recorded at harvest, which was in mid-August. The following formula was then used to estimate the total yield (ton/ha):

$$\:Yield\:(ton/ha)=\frac{yield\:per\:tree\:\left(kg\right)\times\:No.\:of\:trees\:per\:ha}{1000}$$

### Fruit quality

#### Physical fruit quality

At harvest time, twenty fruits were chosen at random from each treatment to gauge the following attributes: Fruit diameter (mm), length (mm), weight (g), juice weight (%), and shape index (length /diameter ratio). Juice weight (JW%) is determined using the following formulas:

$$\:\%\:JW=\frac{JW}{FW}\times\:100$$

where JW is the juice weight (g); FW is the fruit weight (g).

$${\text{Shape Index}}={\text{Fruit height (mm)/Fruit diameter (mm)}}.$$

#### Chemical characteristics

To find the total soluble solids percentage (TSS%), a hand refractometer was employed. In accordance with AOAC^27^, total acidity (%) was calculated. Vitamin C was calculated using the AOAC^27^ as (mg) ascorbic acid/100 ml of juice.

### Plant analysis

Total elements such as nitrogen, phosphorus, and potassium were determined in dried leaves of acid lime samples after digestion with a mixture of H_2_SO_4_-H_2_O_2_^28^. Total nitrogen in all digestive samples was estimated by the Micro-Kjeldahl method, and phosphorus was measured colorimetrically by the phosphomolybdic acid method in a sulfuric acid system^24^. Potassium was analyzed by flame photometry.

### Statistical analysis

A randomized complete block design including three replicates was used to arrange all the experiment’s treatments. Statistics 8.1 software (Analytical Software, 2005) was used to statistically analyze the data using the one-way ANOVA. Using the Tukey test at *p* ≤ 0.05, means were compared for significant differences. The data were shown as means plus standard deviations.

## Results

### Nutrient soil availability

In the first season, amending PL soil with co-applied zeolite significantly improved available nitrogen in soil compared to the 100%MNF treatment, whereas in the second season, the differences between treatments showed a non-significant increase compared to the 50%MNF treatment (Table 2). The concentration of soil available nitrogen in the first season increased from 26.98 mg kg^− 1^ for 100%MNF to 44.39, 36.55, 60.05, and 74.41 mg kg^− 1^ for 50%MNF, BPL + 50%MNF, and ZPL + 50%MNF, respectively. Significant increase in soil available phosphorus in the first season when applying PL incorporated with biochar compared to the 50%MNF, 100%MNF, and PL treatments. whereas in the second season, the differences between treatments showed a non-significant decrease compared to the 50%MNF treatment (Table 2). The concentration of soil available phosphorus in the first season increased from 2.34 mg kg^− 1^ for 100%MNF to 6.71, 6.70, 18.45, and 12.75 mg kg^− 1^ for 50%MNF, BPL + 50%MNF, and ZPL + 50%MNF, respectively. Soil available potassium significantly increased in the first season with applying PL incorporated with biochar in comparison with the rest of the treatments under study. Whereas in the second season, the differences between treatments under study showed a non-significant effect (Table 2). The concentration of soil available potassium in the first season increased from 57.00 mg kg^− 1^ for PL + 50%MNF to 66.18, 66.56, 95.45, and 83.27 mg kg^− 1^ for 50%MNF, 100%MNF, BPL + 50%MNF, and ZPL + 50%MNF, respectively.

**Table 2:** Effect of biochar- and zeolite-amended poultry litter on nutrient availability in calcareous sandy soil after the harvest of the acid lime fruits (data were mean ± standard deviation, *n* = 3).

Treatments	Available nutrient content (mg kg^− 1^)					
	Nitrogen		Phosphorus		Potassium	
	First season (2023)	Second season (2024)	First season (2023)	Second season (2024)	First season (2023)	Second season (2024)
50%MNF	44.39 ± 10.44^ab^	30.93 ± 3.71^a^	6.71 ± 3.03^b^	12.44 ± 6.44^a^	66.18 ± 1.15^b^	102.72 ± 9.76^a^
100%MNF	26.98 ± 17.77^b^	39.59 ± 12.86^a^	2.34 ± 0.07^b^	8.87 ± 2.46^a^	66.56 ± 10.33^b^	113.62 ± 17.21^a^
PL + 50%MNF	36.55 ± 10.44^b^	39.59 ± 2.47^a^	6.70 ± 4.64^b^	8.71 ± 3.52^a^	57.00 ± 1.15^b^	86.33 ± 18.61^a^
BPL + 50%MNF	60.05 ± 18.28^ab^	38.35 ± 3.71^a^	18.45 ± 6.11^a^	7.73 ± 0.14^a^	95.45 ± 12.05^a^	75.94 ± 5.93^a^
ZPL + 50%MNF	74.41 ± 6.53^a^	35.47 ± 14.29^a^	12.75 ± 0.50^ab^	7.96 ± 0.23^a^	83.27 ± 11.51^ab^	105.59 ± 1.91^a^

Different superscript lowercase letters within each column indicate significant differences among means of treatments, whereas means sharing the same letter are not significantly different according to Tukey’s honestly significant difference (HSD) test at *p* ≤ 0.05. MNF: mineral nitrogen fertilizer; PL: poultry litter; B: biochar; Z: zeolite.

### Vegetative growth parameters

It is quite evident, as shown in Table 3, that all the evaluated growth measurements (average length of branches, number of leaves per branch, leaf area, and leaf total chlorophyll) of acid lime in response to diverse mineral and organic fertilization treatments, either applied alone or in combinations, were investigated. There was a significant difference (*P* < 0.05) among various fertilization treatments. In the first season, the applications of 100%MNF, PL + 50%MNF, BPL + 50%MNF, and ZPL + 50%MNF to this soil significantly increased the average length of branches, number of leaves per branch, and leaf area of acid lime compared with the 50%MNF treatment. Moreover, the leaf total chlorophyll significantly increased with the addition of 100%MNF, BPL + 50%MNF, and ZPL + 50%MNF compared to 50%MNF treatment. Likewise, there were no significant differences between the 100%MNF, BPL + 50%MNF, and ZPL + 50%MNF treatments in terms of their influence on vegetative growth parameters. In the second season, there were no significant differences between all the treatments under study in their effect on the studied vegetative growth parameters (Table 3).

**Table 3:** Effect of biochar or zeolite amended poultry litter on shoot length, leaves number, leaf area, and chlorophyll of acid lime trees grown in calcareous sandy soil (Data were mean ± standard deviation, *n* = 3).

Treatments	Shoot length (cm)		Leaves number		Leaf area (cm^2^)		Chlorophyll (SPAD)	
	First season (2023)	Second season (2024)	First season (2023)	Second season (2024)	First season (2023)	Second season (2024)	First season (2023)	Second season (2024)
50%MNF	42.44 ± 0.46^c^	44.34 ± 0.34^a^	23.33 ± 0.88^c^	25.05 ± 1.02^a^	13.03 ± 0.08^c^	14.22 ± 0.07^a^	26.43 ± 0.04^c^	25.05 ± 0.04^a^
100%MNF	48.23 ± 0.36^a^	48.02 ± 0.76^a^	34.87 ± 0.68^a^	35.09 ± 0.69^a^	17.98 ± 0.11^a^	16.77 ± 1.02^a^	44.61 ± 0.07^a^	34.68 ± 0.03^a^
PL + 50%MNF	45.98 ± 1.00^b^	50.04 ± 0.55^a^	27.56 ± 1.02^b^	36.88 ± 0.65^a^	15.99 ± 0.99^b^	18.05 ± 1.00^a^	31.07 ± 0.03^bc^	37.87 ± 0.06^a^
BPL + 50%MNF	47.89 ± 0.63^a^	48.96 ± 0.35^a^	34.55 ± 0.57^a^	36.07 ± 0.34^a^	17.22 ± 0.34^ab^	17.65 ± 0.98^a^	41.76 ± 0.07^ab^	36.98 ± 0.24^a^
ZPL + 50%MNF	47.55 ± 0.21^ab^	46.12 ± 0.28^a^	34.32 ± 0.44^a^	30.43 × 0.76^a^	16.43 ± 0.32^ab^	15.05 ± 0.55^a^	37.54 ± 0.00^ab^	31.50 ± 0.01^a^

Different superscript lowercase letters within each column indicate significant differences among means of treatments, whereas means sharing the same letter are not significantly different according to Tukey’s honestly significant difference (HSD) test at *p* ≤ 0.05.

*MNF* mineral nitrogen fertilizer, *PL* poultry litter, *B* biochar, *Z* zeolite.

### Yield components

**Fig. 1:**
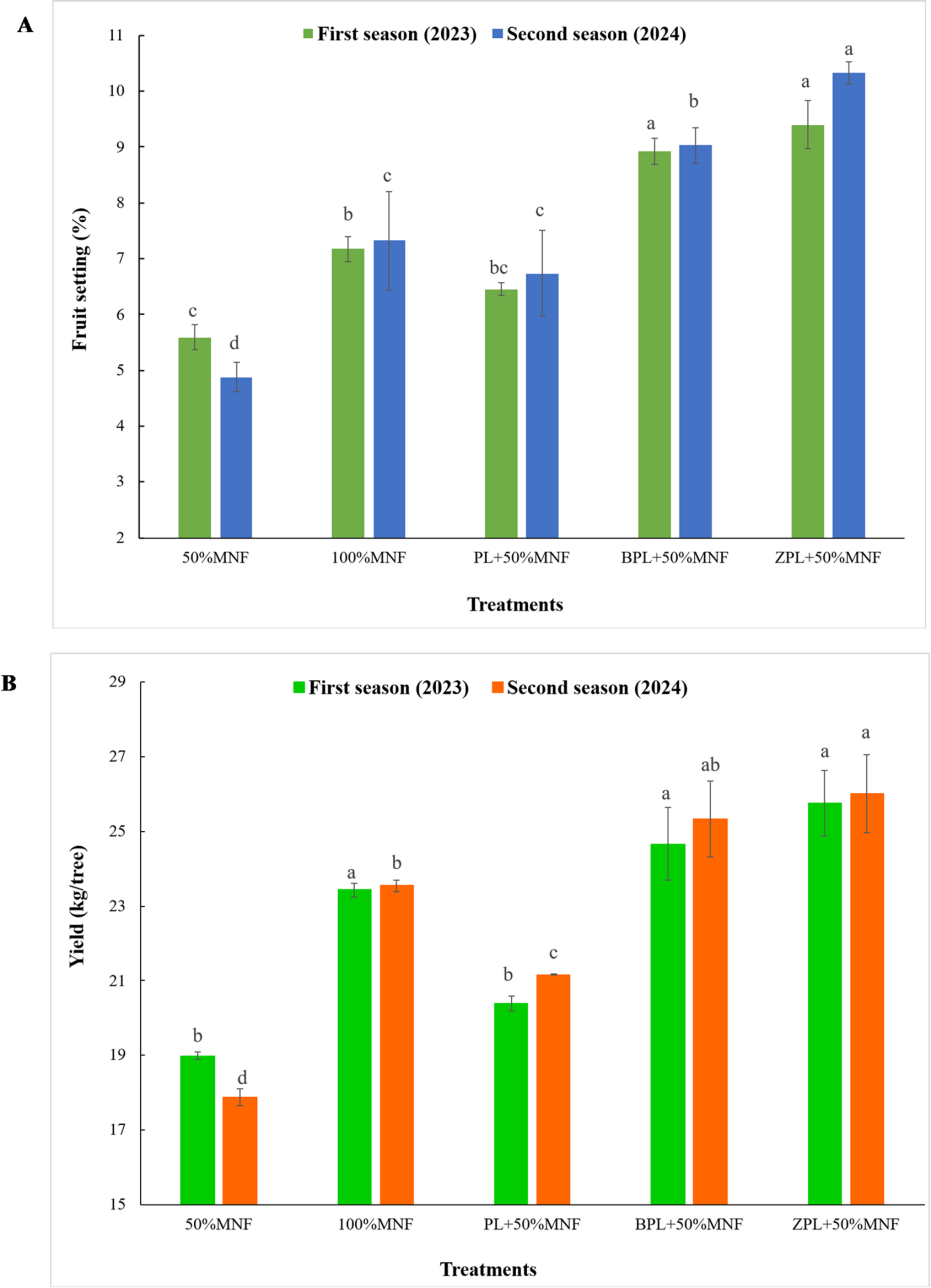
Effect of biochar- and zeolite-amended poultry litter on fruit setting (**A**) and yield (kg/tree) (**B**) of acid lime trees grown in calcareous sandy. Each value represents the average of three replicates. Different lowercase letters on each bar indicate significant differences among means of treatments, whereas means sharing the same letter are not significantly different according to Tukey’s honestly significant difference (HSD) test at *p* ≤ 0.05. Vertical bars refer to the standard deviation of the mean (*n* = 3 replicates). MNF: mineral nitrogen fertilizer; PL: poultry litter; B: biochar; Z: zeolite.

**Fig. 2:**
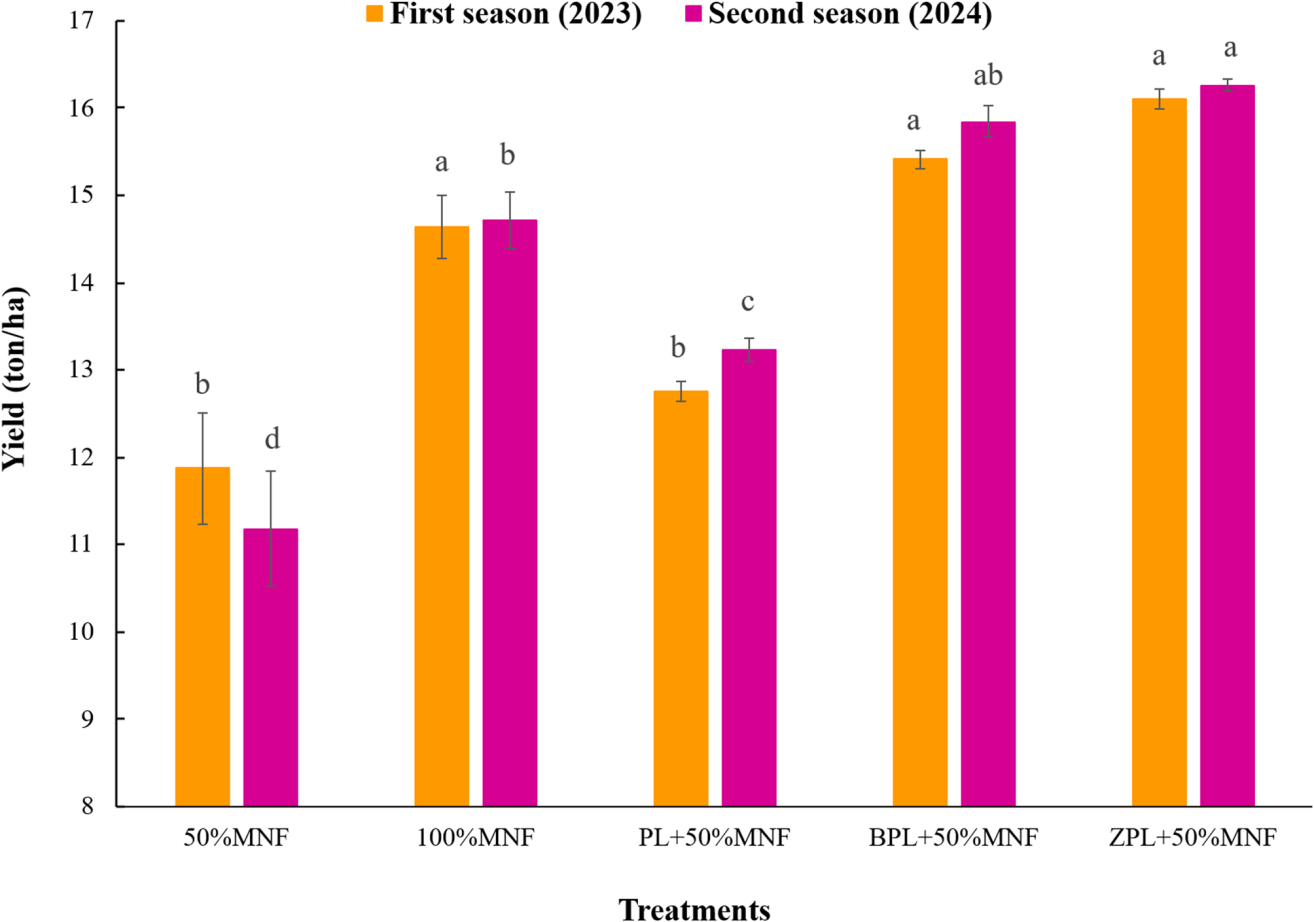
Effect of biochar- and zeolite-amended poultry litter on yield (ton/ha) of acid lime trees grown in calcareous sandy soil. Each value represents the average of three replicates. Different lowercase letters on each bar indicate significant differences among means of treatments, whereas means sharing the same letter are not significantly different according to Tukey’s honestly significant difference (HSD) test at *p* ≤ 0.05. Vertical bars refer to the standard deviation of the mean (*n* = 3 replicates). MNF: mineral nitrogen fertilizer; PL: poultry litter; B: biochar; Z: zeolite.

Compared to 50%MNF treatment, all investigated treatments had a significant effect on yield and fruit setting percentage during each of the two experimental seasons, except for PL + 50%MNF in the first season (Figs. 1A and B, and 2), which displays the impacts of various treatments on yield components (fruit setting percentage, yield (kg/tree), and yield (ton/ha). The results obtained in this study revealed that the acid lime fertilized with BPL + 50%MNF and ZPL + 50%MNF treatments showed the best treatments in terms of fruit setting percentage and the highest yield per tree or ton/ha compared to the rest of the treatments in two seasons. The lowest values of yield components (fruit setting percentage, yield (kg/tree), and yield (ton/ha) in this study were recorded for 50%MNF during each of the two experimental seasons. In the first season, the fruit setting percentage increased from 5.59% for 50%MNF to 7.17, 6.45, 8.92, and 9.40% for 100%MNF, PL + 50%MNF, BPL + 50%MNF, and ZPL + 50%MNF treatments, respectively (Fig. 1A). The yield of acid lime (kg/tree) increased from 18.99 kg/tree (50%MNF) to 23.43, 20.40, 24.66, and 25.75 kg/tree for 100%MNF, PL + 50%MNF, BPL + 50%MNF, and ZPL + 50%MNF treatments, respectively (Fig. 1b). Also, the yield of acid lime (ton/ha) increased from 11.87 ton/ha (50%MNF) to 14.64, 12.75, 15.41, and 16.10 ton/ha for 100%MNF, PL + 50%MNF, BPL + 50%MNF, and ZPL + 50%MNF treatments, respectively (Fig. 2). Moreover, in the second season, the fruit setting percentage increased from 4.88% for 50%MNF to 7.32, 6.73, 9.03, and 10.33% for 100%MNF, PL + 50%MNF, BPL + 50%MNF, and ZPL + 50%MNF treatments, respectively. The yield of acid lime (kg/tree) increased from 17.88 kg/tree (50%MNF) to 23.54, 21.16, 25.34, and 26.01 kg/tree for 100%MNF, PL + 50%MNF, BPL + 50%MNF, and ZPL + 50%MNF treatments, respectively. Also, the yield of acid lime (ton/ha) increased from 11.11 ton/ha (50%MNF) to 14.71, 13.23, 15.84, and 16.26 ton/ha for 100%MNF, PL + 50%MNF, BPL + 50%MNF, and ZPL + 50%MNF treatments, respectively. Generally, the effectiveness of treatments in improving the yield of acid lime (ton/ha) in both seasons was in the order of ZPL + 50%MNF > BPL+50%MNF > 100%MNF > PL + 50%MNF > 50%MNF (Fig. 2). The differences between ZPL + 50%MNF and BPL + 50%MNF were nonsignificant in all yield component parameters.

### Fruit properties

#### Physical fruit properties

**Fig. 3:**
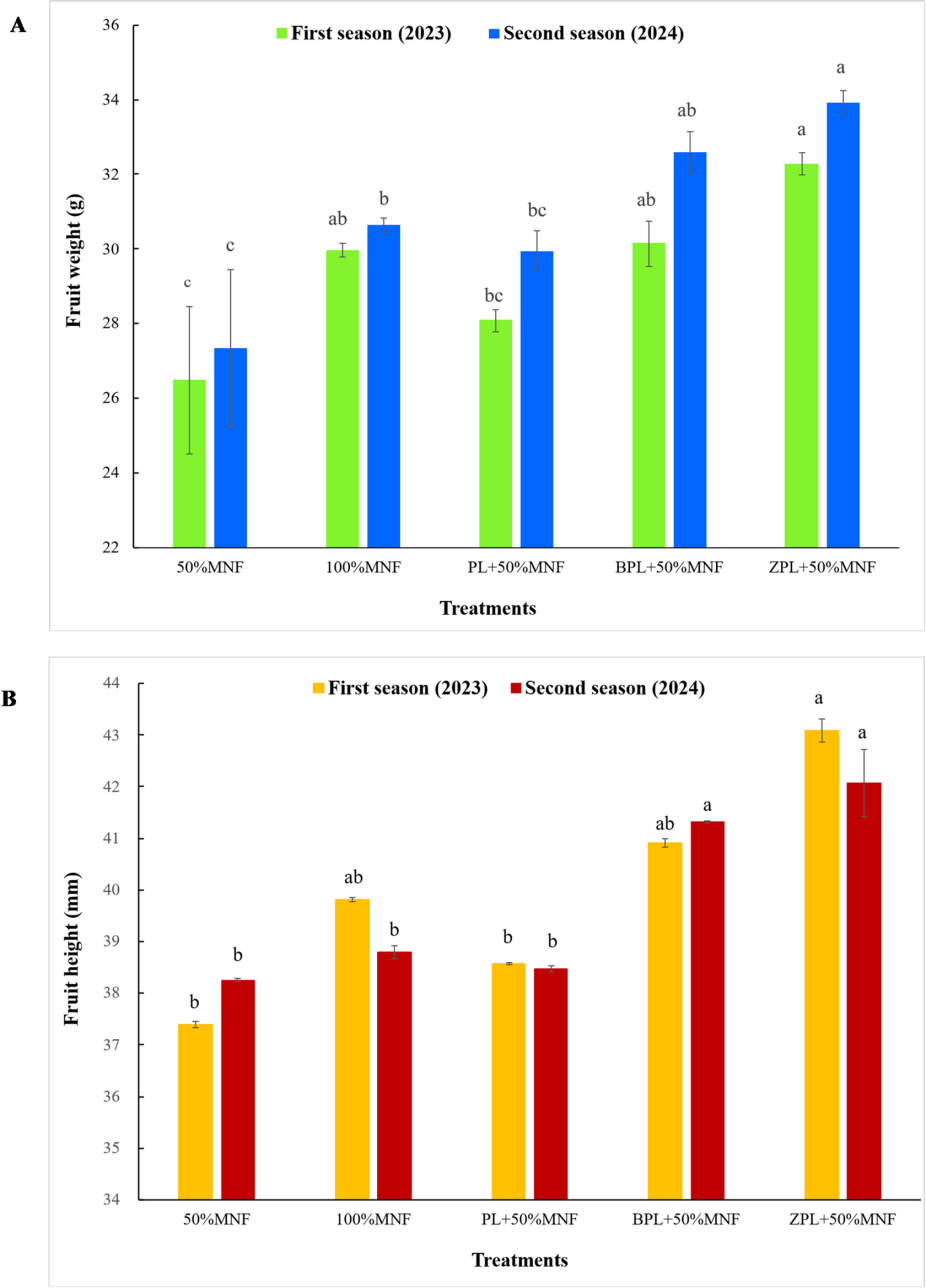
Effect of biochar- and zeolite-amended poultry litter on fruit weight (**A**) and fruit height (**B**) of acid lime trees grown in calcareous sandy soil. Each value represents the average of three replicates. Different lowercase letters on each bar indicate significant differences among means of treatments, whereas means sharing the same letter are not significantly different according to Tukey’s honestly significant difference (HSD) test at *p* ≤ 0.05. Vertical bars refer to the standard deviation of the mean (*n* = 3 replicates). MNF: mineral nitrogen fertilizer; PL: poultry litter; B: biochar; Z: zeolite.

**Fig. 4:**
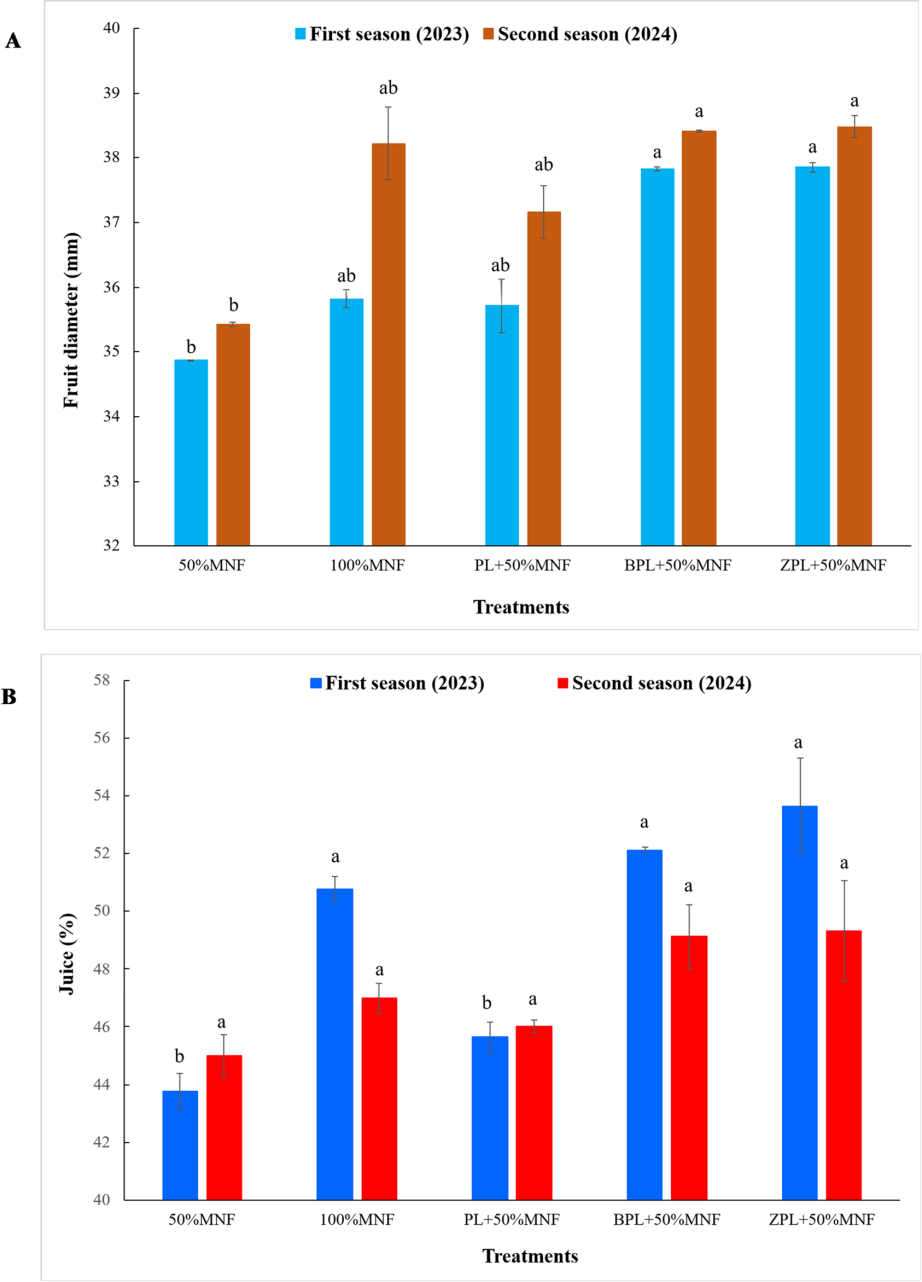
Effect of biochar- and zeolite-amended poultry litter on fruit diameter (**A**) and juice (**B**) of acid lime trees grown in calcareous sandy soil. Each value represents the average of three replicates. Different lowercase letters on each bar indicate significant differences among means of treatments, whereas means sharing the same letter are not significantly different according to Tukey’s honestly significant difference (HSD) test at *p* ≤ 0.05. Vertical bars refer to the standard deviation of the mean (*n* = 3 replicates). MNF: mineral nitrogen fertilizer; PL: poultry litter; B: biochar; Z: zeolite.

Data, which was graphically represented with (Figs. 3A,B and 4A,B), showed the effect of different treatments on the physical properties of acid lime fruit during the 2023 and 2024 seasons. It was obvious from the data that the results followed a similar trend during the two studied seasons. Results showed that the fruit weight of acid lime significantly increased under applying 100%MNF, BPL + 50%MNF, and ZPL + 50%MNF treatments during two seasons. However, adding PL + 50%MNF treatment caused a nonsignificant increase in fruit weight, also during the two seasons (Fig. 3A). The fruit weight increased from 26.48 g for 50%MNF treatment to 29.96, 28.08, 30.14, and 32.27 g for 100%MNF, PL + 50%MNF, BPL + 50%MNF, and ZPL + 50%MNF treatments, respectively, in the first season. However, the fruit weight increased from 27.34 g for 50%MNF treatment to 30.63, 29.95, 32.60, and 33.92 g for 100%MNF, PL + 50%MNF, BPL + 50%MNF, and ZPL + 50%MNF treatments, respectively, in the second season. The lowest values of fruit weight in this study were recorded for 50% MNF during both experimental seasons. The highest values of fruit weight were observed when adding the BPL + 50%MNF and ZPL + 50%MNF treatments during two seasons (Fig. 3A). In the first season, the addition of ZPL + 50%MNF treatment alone led to a significant increase in fruit height, while the other treatments led to an insignificant increase compared to 50%MNF treatment. However, in the second season, the addition of BPL + 50%MNF and ZPL + 50%MNF treatments alone led to a significant increase in fruit height. In contrast, the other treatments resulted in an insignificant increase compared to the 50% MNF treatment. The results obtained in this study revealed that the acid lime fertilized with BPL + 50%MNF and ZPL + 50%MNF treatments showed significantly increased fruit diameter compared to the 50% MNF treatment in both seasons; however, the other treatments resulted in an insignificant increase compared to the 50% MNF treatment in both seasons. The fruit diameter increased from 34.87 mm (50% MNF) to 35.82, 35.71, 37.83, and 37.85 mm for 100%MNF, PL + 50%MNF, BPL + 50%MNF, and ZPL + 50%MNF treatments, respectively, in the first season. Moreover, the fruit diameter increased from 35.42 mm for 50% MNF treatment to 38.22, 37.16, 38.41, and 38.48 mm for 100%MNF, PL + 50%MNF, BPL + 50%MNF, and ZPL + 50%MNF treatments, respectively, in the second season. Significant increase in the juice weight (%) with the application of 100%MNF, BPL + 50%MNF, and ZPL + 50%MNF treatments compared to the control treatment in the first season. However, in the second season, there are no significant differences between all the treatments under study. Juice weight in the first season increased from 43.76% for 50% MNF treatment to 50.77, 45.64, 52.11, and 53.65% for 100%MNF, PL + 50%MNF, BPL + 50%MNF, and ZPL + 50%MNF treatments, respectively.

#### Chemical constituents of juice

Data concerning some chemical properties (percentage of total soluble solids, total acidity, and vitamin C content (mg/100 ml juice) of lime juice are demonstrated in Table 4. It’s obvious that the results followed a similar trend during the two studied seasons. There were significant differences between the treatments. Compared to the 50% MNF treatment, the applications of 100%MNF, PL + 50%MNF, BPL + 50%MNF, and ZPL + 50%MNF treatments to acid lime led to a significant increase in total soluble solids and total acidity in both seasons. In the first season, vitamin C content in juice increased significantly with adding BPL + 50%MNF and ZPL + 50%MNF treatments, but adding PL + 50%MNF caused a significant decrease in vitamin C content in juice compared to the 50%MNF treatment. In the second season, the additions of 100%MNF, PL + 50%MNF, BPL + 50%MNF, and ZPL + 50%MNF treatments caused a significant increase in vitamin C content in juice compared to the 50%MNF treatment. Generally, the highest values of total soluble solids, total acidity, and vitamin C content were observed when adding the BPL + 50%MNF and ZPL + 50%MNF treatments during two seasons (Table 4).

**Table 4:** Effect of biochar- and zeolite-amended poultry litter on TSS, total acidity, and vitamin C of acid lime trees growing in calcareous sandy soil (data were mean ± standard deviation, *n* = 3).

Treatments	TSS (%)		Total acidity (%)		Vitamin C (mg/100 ml juice)	
	First season (2023)	Second season (2024)	First season (2023)	Second season (2024)	First season (2023)	Second season (2024)
50%MNF	8.11 ± 0.10^d^	8.04 ± 0.13^d^	7.19 ± 0.10^d^	7.15 ± 0.15^e^	50.45 ± 0.82^c^	42.47 ± 0.96^d^
100%MNF	9.01 ± 0.20^b^	8.90 ± 0.15^c^	8.03 ± 0.15^b^	7.99 ± 0.17^c^	49.92 ± 0.55^c^	56.09 ± 0.63^c^
PL + 50%MNF	8.46 ± 0.15^c^	8.80 ± 0.17^c^	7.44 ± 0.20^c^	7.81 ± 0.12^d^	45.32 ± 0.20^d^	55.22 ± 0.27^c^
BPL + 50%MNF	9.15 ± 0.15^b^	9.87 ± 0.16^b^	8.12 ± 0.02^b^	8.83 ± 0.15^b^	55.42 ± 0.75^b^	58.70 ± 0.44^b^
ZPL + 50%MNF	9.64 ± 0.10^a^	10.30 ± 0.15^a^	8.70 ± 0.10^a^	9.32 ± 0.15^a^	57.33 ± 0.36^a^	60.01 ± 0.30^a^

TSS: total soluble solids. Different superscript lowercase letters within each column indicate significant differences among means of treatments, whereas means sharing the same letter are not significantly different according to Tukey’s honestly significant difference (HSD) test at *p* ≤ 0.05.

*MNF* mineral nitrogen fertilizer, *PL* poultry litter, *B* biochar, *Z* zeolite.

### Nutrient content in acid lime leaves

The nitrogen content in acid lime leaves increased significantly with the application of 100%MNF, BPL + 50%MNF, and ZPL + 50%MNF treatments compared to 50%MNF and PL + 50%MNF treatments in the first season (Table 5). The nitrogen content in acid lime leaves increased from 15.04 g kg^− 1^ for 50%MNF to 19.81, 17.22, 19.73, and 19.69 g kg^− 1^ for 100%MNF, PL + 50%MNF, BPL + 50%MNF, and ZPL + 50%MNF, respectively. In the second season, the differences between treatments under study showed a non-significant effect (Table 5). In the first season, there are no significant differences in phosphorus content in acid lime leaves between the treatments. Phosphorus content in acid lime leaves increased significantly when PL + 50%MNF was added to the soil under study, compared to the rest of the treatments in the second season. Phosphorus content in acid lime leaves increased from 0.78 g kg^− 1^ for 50%MNF to 0.84, 1.30, 0.79, and 0.97 g kg^− 1^ for 100%MNF, PL + 50%MNF, BPL + 50%MNF, and ZPL + 50%MNF, respectively. In the first season, there are no significant differences in potassium content in acid lime leaves between the treatments. Potassium content in acid lime leaves increased significantly when ZPL + 50%MNF was added to the soil under study, compared to the rest of the treatments in the second season. Potassium content in acid lime leaves increased from 7.42 g kg^− 1^ for PL + 50%MNF to 12.49, 10.79, 10.95, and 13.88 g kg^− 1^ for 50%MNF, 100%MNF, BPL + 50%MNF, and ZPL + 50%MNF, respectively (Table 5).

**Table 5:** Effect of biochar- and zeolite-amended poultry litter on nutrient content in leaves of acid lime trees (Data were mean ± standard deviation, *n* = 3).

Treatments	Nutrient content in leaves (g kg^− 1^)					
	Nitrogen		Phosphorus		Potassium	
	First season (2023)	Second season (2024)	First season (2023)	Second season (2024)	First season (2023)	Second season (2024)
50%MNF	15.04 ± 0.74^c^	17.55 ± 0.35^a^	0.88 ± 0.08^a^	0.78 ± 0.06^b^	10.49 ± 0.74^a^	12.49 ± 0.54^b^
100%MNF	19.81 ± 0.27^a^	19.44 ± 0.63^a^	1.12 ± 0.09^a^	0.84 ± 0.16^b^	10.42 ± 0.97^a^	10.79 ± 0.37^c^
PL + 50%MNF	17.22 ± 0.17^b^	20.53 ± 0.66^a^	0.97 ± 0.06^a^	1.30 ± 0.02^a^	8.64 ± 0.49^a^	7.42 ± 0.07^d^
BPL + 50%MNF	19.73 ± 0.35^a^	20.48 ± 1.72^a^	0.89 ± 0.02^a^	0.79 ± 0.15^b^	9.73 ± 2.06^a^	10.95 ± 0.42^c^
ZPL + 50%MNF	19.69 ± 0.86^a^	19.33 ± 2.42^a^	1.12 ± 0.40^a^	0.97 ± 0.25^ab^	9.82 ± 0.26^a^	13.88 ± 0.90^a^

Different superscript lowercase letters within each column indicate significant differences among means of treatments, whereas means sharing the same letter are not significantly different according to Tukey’s honestly significant difference (HSD) test at *p* ≤ 0.05. MNF: mineral nitrogen fertilizer; PL: poultry litter; B: biochar; Z: zeolite.

## Discussion

To enhance the sustainability and productivity of modern agriculture, it is crucial to improve the efficiency of chemical fertilizers through natural, low-impact strategies without compromising crop productivity or tree health. Biochar combined with poultry manure and chemical fertilizers enhanced the water-holding capacity, organic matter content, and phosphorus availability in calcareous sandy soil^29^. Also, the co-application of biochar with poultry litter caused a significant increase in total nitrogen and exchangeable potassium in sandy soil^30^. Combined application of poultry litter and biochar significantly increased total nitrogen in the soil compared to the sole application of either biochar or poultry litter^31^. The high surface area, porous structure, and ash content of biochar, along with the rapid nutrient mineralization from poultry litter, contribute to improved soil properties. Moreover, the capacity of biochar to retain nutrients in the soil helps reduce nutrient leaching from the poultry litter^31^. The combined application of biochar and mineral fertilizers caused a significant increase in total available nitrogen and available potassium in the soil^32^. The use of zeolite mixed with chemical fertilizer had a positive effect on soil structure, as it enhanced the fine fraction content, which in turn improved soil fertility due to its ability to retain nutrients, microorganisms, and water, as well as enhance plant nutrition^33^. The benefits of using the prepared zeolite-based fertilizer are low cost due to simple processing from local resources, reducing fertilizer consumption, and retail of high possibility for amending the sandy soil^33^. Zeolite can be used as a soil conditioner in agriculture to improve the soil’s physical and chemical properties, including infiltration rate, saturated hydraulic conductivity, water-holding capacity, and cation exchange capacity^11^.

The results previously discussed are supported by various researchers, who have indicated that chicken manure, an organic fertilizer rich in essential nutrients such as nitrogen, phosphorus, and potassium, is a valuable addition to improve soil quality indicators. Applying biochar with chemical fertilizers in sandy soil increased the yield, fruit diameter, peel thickness, fruit weight, vitamin C, and total soluble solids of lemon during two growth seasons. These results are attributed to applying biochar to the sandy soil, which led to enhancing water-holding capacity within the root zone, hence improving the efficiency of adding irrigation water. Also, biochar has increased nutrient availability^34^. Co-applications of biochar with chemical fertilizers resulted in about 1.5-fold higher fruit yield of pomelo (*Citrus grandis* Osbeck) than that of chemical fertilizers alone, which is attributed to improving soil fertility. Moreover, applying biochar with chemical fertilizer significantly improved the farmer’s livelihood by enhancing profit compared with using chemical fertilizers alone^35^. Generally, applying biochar to citrus orchards increases the availability of essential nutrients such as nitrogen, phosphorus, and potassium, thereby enhancing plant growth and fruit quality as well as disease suppression. Citrus orchard soils that have been amended with biochar can better retain water and lose less water to leaching^36^. The green biomass of barley plants improved significantly as a result of applying biochar mixed with poultry manure in the presence of chemical fertilizers^29^. The combined application of biochar and chicken manure significantly improved pea growth and yield compared to the control^37^. Biochar has been used as a sustainable amendment to mitigate environmental risks, improve plant growth, and soil properties^38^. Application of biochar in combination with chemical fertilizers improved the crop yield because applying biochar increases total soil porosity, leading to a reduction in bulk density and improvements in soil aggregation and water-holding capacity. Although biochar itself contains considerable amounts of plant nutrients^39^. Also, adding biochar with chemical fertilizers improves the growth and yield of acid lime, which is attributed to biochar addition to the sandy soil in drylands due to an increase in water holding capacity and nutrient retention^40^. The integration of biochar with inorganic fertilizer and organic manure application can improve the productivity of maize and provide more sustainable input of nitrogen and phosphorus to soil^41^. The application of poultry manure mixed with biochar to existing chemical fertilizers in calcareous sandy soil significantly increased the uptake and content of nitrogen and phosphorus in barley plants compared to unamended soil^29^. In our study, significant differences in P and K content in leaves of acid lime trees during the second season may be attributed to the cumulative effect of amendments and seasonal variability. Abdel Monsef et al.^42^ demonstrated that the foliar application of camel manure tea or duck manure tea, combined with the recommended chemical fertilizers, enhanced the yield of acid lime fruits grown in sandy calcareous soil under a drip irrigation system, reaching 13.60 and 13.90 ton/ha (equivalent to 21.83 and 22.17 kg/tree) for camel manure tea, and 14.00 and 15.10 ton/ha (equivalent to 22.40 and 24.23 kg/tree) for duck manure tea during the first and second seasons, respectively. Compared with the findings of Abdel Monsef et al.^42^, who conducted their study under similar environmental and soil conditions, our results showed higher productivity, suggesting that the treatments applied in the present study were more effective in improving productivity.

The combined application of zeolite and organic matter enhances ramie plant growth by increasing plant height and leaf number. This improvement is attributed to the positive effects of zeolite and organic matter on soil aggregation, as well as their ability to enhance soil nutrient and water retention capacity^43^. Zeolites are well characterized for their ability to retain and gradually release macronutrients, micronutrients, and fertilizers. Their potential application in agricultural systems is evident, as zeolites offer significant promise as a sustainable amendment for directly enhancing agroecosystems^5^. The combined application of chemical fertilizers and zeolite improved physiological and biochemical performance, as reflected by sustained increases in relative water content, stomatal conductance, and net photosynthetic rate, along with reduced indicators of oxidative stress during periods of heightened climatic stress^44^. Soil application of zeolite in olive growing increased nitrogen retention time in the soil, enabling more efficient uptake by plants. The application of zeolite in olive cultivation enables a reduction in fertilizer input of up to 50% while enhancing nitrogen use efficiency, thereby delivering significant environmental and economic benefits^45^. Natural zeolite application to the sandy loam soil increased cumulative yield, fruit weight, flesh rate, and geometric mean diameter^46^, because the combined application of zeolite, manure, and chemical fertilizers improved soil chemical properties and enhanced the retention of nitrogen and potassium within the root zone, thereby ensuring their availability for plant uptake when needed^47^.

The integration of poultry litter with biochar or zeolite as a soil amendment, combined with a 50% reduction in mineral nitrogen fertilizer, resulted in a noticeable increase in acid lime yield. This improvement can be attributed to enhanced nutrient availability in the soil, as both biochar and zeolite are known to reduce nutrient losses through leaching and volatilization. Moreover, the combined application likely improved soil physical and chemical properties, promoting better root development and nutrient uptake. Overall, this strategy demonstrates the potential to sustain or enhance lemon productivity while substantially reducing dependence on mineral nitrogen fertilizers, offering both agronomic and environmental benefits. This approach focuses on reducing the use of chemical fertilizers by partially substituting mineral nitrogen with poultry litter amended with biochar or zeolite, allowing a 50% reduction in mineral nitrogen application while increasing acid lime yield.

## Conclusions

This study addressed a critical knowledge gap in nitrogen management for lemon orchards by evaluating the use of biochar- and zeolite-amended poultry litter with a 50% reduction in mineral nitrogen fertilizer. The addition of biochar- and zeolite-amended poultry litter, with half the amount of chemical nitrogen fertilizer, to calcareous sandy soil has the potential to increase the yield of acid lime, reduce dependence on chemical nitrogen fertilizer, and improve soil quality and resilience. The highest values of total soluble solids, total acidity, and vitamin C content were observed when adding the biochar- and zeolite-amended poultry litter treatments during two seasons. The partial substitution of mineral nitrogen fertilizer with poultry litter amended with biochar or zeolite proved to be an effective strategy for reducing chemical fertilizer use. A 50% reduction in mineral nitrogen application not only maintained but also enhanced acid lime yield, indicating reduced nutrient losses. This integrated approach offers a sustainable and environmentally friendly alternative to conventional fertilization practices in acid lime production systems. Consequently, to improve soil fertility and obtain the highest yield and quality of acid lime, it is recommended to add 10 kg of poultry litter amended with biochar or zeolite and 0.5 kg N as ammonium nitrate per acid lime tree in calcareous sandy soil under a drip irrigation system.

## Data Availability

The datasets used or analyzed during the current study are available from the corresponding author upon reasonable request.
